# Detecting Falls with Wearable Sensors Using Machine Learning Techniques

**DOI:** 10.3390/s140610691

**Published:** 2014-06-18

**Authors:** Ahmet Turan Özdemir, Billur Barshan

**Affiliations:** 1. Department of Electrical and Electronics Engineering, Erciyes University, Melikgazi, Kayseri TR-38039, Turkey; E-Mail: aturan@erciyes.edu.tr; 2. Department of Electrical and Electronics Engineering, Bilkent University, Bilkent, Ankara TR-06800, Turkey

**Keywords:** fall detection, activities of daily living, wearable motion sensors, machine learning, pattern classification, feature extraction and reduction

## Abstract

Falls are a serious public health problem and possibly life threatening for people in fall risk groups. We develop an automated fall detection system with wearable motion sensor units fitted to the subjects' body at six different positions. Each unit comprises three tri-axial devices (accelerometer, gyroscope, and magnetometer/compass). Fourteen volunteers perform a standardized set of movements including 20 voluntary falls and 16 activities of daily living (ADLs), resulting in a large dataset with 2520 trials. To reduce the computational complexity of training and testing the classifiers, we focus on the raw data for each sensor in a 4 s time window around the point of peak total acceleration of the waist sensor, and then perform feature extraction and reduction. Most earlier studies on fall detection employ rule-based approaches that rely on simple thresholding of the sensor outputs. We successfully distinguish falls from ADLs using six machine learning techniques (classifiers): the k-nearest neighbor (*k*-NN) classifier, least squares method (LSM), support vector machines (SVM), Bayesian decision making (BDM), dynamic time warping (DTW), and artificial neural networks (ANNs). We compare the performance and the computational complexity of the classifiers and achieve the best results with the *k*-NN classifier and LSM, with sensitivity, specificity, and accuracy all above 99%. These classifiers also have acceptable computational requirements for training and testing. Our approach would be applicable in real-world scenarios where data records of indeterminate length, containing multiple activities in sequence, are recorded.

## 1. Introduction

With the world's aging population, health-enabling technologies and ambulatory monitoring of the elderly has become a prominent area of multi-disciplinary research [1,2]. Rapidly developing technology has made mobile and wireless devices part of daily life. An important aspect of context-aware systems is recognizing, interpreting, and monitoring the basic activities of daily living (ADLs) such as standing, sitting, lying down, walking, ascending/descending stairs, and most importantly, emergent events such as falls. If a sudden change in the center of mass of the human body results in a loss of balance, the person falls. The World Health Organization defines falls as involuntary, unexpected, and uncontrollable events resulting in a person impacting and coming to rest on the ground or at a lower level [3].

Falls need to be considered within the same framework as ADLs since they typically occur unexpectedly while performing daily activities. Falls are a public health problem and a health threat, especially for adults of age 65 and older [4]. Statistics indicate that one in every three adults of age 65 or older experiences at least one fall every year. Besides the elderly, children, disabled individuals, workers, athletes, and patients with visual, balance, gait, orthopedic, neurological, and psychological disorders also suffer from falls. The intrinsic factors associated with falls are aging, mental impairment, neurological and orthopedic diseases, vision and balance disorders. The extrinsic factors are multiple drug usage, slippery floors, poor lighting, loose carpets, handrails near bathtubs and toilets, electric or power cords, clutter and obstacles on stairways [5]. Although some of the extrinsic risk factors can be eliminated by taking necessary precautions, intrinsic factors are not readily eliminated and falls cannot be completely prevented. Since the consequences of falls can be serious and costly, falls should be detected reliably and promptly to reduce the occurrence of related injuries and the costs of healthcare. Accurate, reliable, and robust fall detection algorithms that work in real time are essential.

Monitoring people in fall risk groups should occur without intruding on their privacy, restricting their independence, or degrading their quality of life. User-activated fall detection systems do not have much practical usage. Fall detection systems need to be completely automated and may rely on multiple sources of sensory information for improved robustness. A commonly used approach is to fix various sensors to the environment, such as cameras, acoustic, pressure, vibration, force, infrared sensors, lasers, Radio Frequency Identification (RFID) tags, inertial sensors and magnetometers [6,7]. Smart environments can be designed through the use of one or more of these sensors in a complementary fashion, usually with high installation cost [8]. Other people or pets moving around may easily confuse such systems and cause false alarms. The main advantage of this approach is that the person at risk does not have to wear or carry any sensors or devices on his body. This approach may be acceptable when the activities of the person are confined to certain parts of a building. However, when the activities performed take place both indoors and outdoors and involve going from one place to another (e.g., riding a vehicle, going shopping, commuting, *etc.*), this approach becomes unsuitable. It imposes restrictions on the mobility of the person since the system operates only in the limited environment monitored by the sensors that are fixed to the environment.

Despite that most earlier studies followed the above approach for monitoring people in the fall risk groups, wearable motion sensors have several advantages. The 1-D signals acquired from the multiple axes of motion sensors are much simpler to process and can directly provide the required 3-D motion information. Unlike visual motion-capture systems that require a free line of sight, inertial sensors can be flexibly used inside or behind objects without occlusion. Because they are light, comfortable, and easy to carry, wearable sensors do not restrict people to a studio-like environment and can operate both indoors and outdoors, allowing free pursuit of activities. The required infrastructure and associated costs of wearable sensors are much lower than smart environments and they do not intrude on privacy. Unlike acoustic sensors, they are not affected by the ambient noise. Wearable sensors are thus suitable for developing automated fall detection systems. In this study, we follow this approach for robust and accurate detection and classification of falls that occur while performing ADLs.

Fall detection is surveyed in [9,10]. Earlier work is fragmented, of limited scope, and not very systematic. The lack of common ground among researchers makes results published so far difficult to compare, synthesize, and build upon in a manner that allows broad conclusions to be reached. Sensor configuration and modality, subject number and characteristics, considered fall types and activities, feature extraction, and acquired signal processing are different in individual studies [11–14]. Although most studies have investigated voluntary (simulated) falls, a limited number of involuntary falls have been recorded in recent studies [15–17]. The latter is a very difficult and time-consuming task [16]. The small number of recorded real-world falls are usually from rare disease populations that cannot be generalized to fall risk groups at large.

Machine learning techniques have been used to distinguish six activities, including falls, using an infrared motion capture system [18]. Studies that use support vector machines are reported in [19,20]. In the latter study, a computer vision based fall recognition system is proposed that combines depth map with normal RGB color information. Better results are achieved with this combination as the depth map reduces the errors and provides more information about the scene. Falls are then recognized and distinguished from ADLs using support vector machines, with accuracy above 95%.

To achieve robust and reliable fall detection and enable comparing different studies, open datasets acquired through standardized experimental procedures are necessary. We found only three works that provide guidelines for fall experiments [21–23] and only one that pursues them [8]. In [23], it is stated that there is no open database for falls and the desirable structure and characteristics of a fall database are described.

Although some commercial devices and patents on fall detection exist, these devices are not satisfactory [22]. The main reasons are the high false alarm rates, high initial and maintenance costs of the devices, and their non-ergonomic nature. Wearable fall detection systems are criticized mainly because people may forget, neglect, or not want to wear them. If they are battery operated, batteries will have to be replaced or recharged from time to time. However, with the advances of the Micro Electro Mechanical Sensors (MEMS) technology, these devices have recently become much smaller, more compact, and less expensive. They can be easily integrated to other available alarm systems in the vicinity or to the accessories that the person carries. The lightness, low power consumption, and wireless use of these devices have eliminated the concerns related to their portability and discomfort. Furthermore, smartphones that usually contain embedded accelerometers are suitable devices for executing fall detection algorithms [24–26].

Through wearable sensors and machine learning techniques, this study aims to robustly and accurately detect falls that occur while performing ADLs. Instead of using simple rule-based algorithms that rely on thresholding the sensory output (as in most earlier works), we employ features of the recorded signals around the point of peak acceleration. To be able to acquire the sufficient amount of data for algorithm development according to the guidelines provided in [23], we limit our study to voluntary (simulated) falls.

The rest of this article is organized as follows: in Section 2, we describe data acquisition and briefly overview the six machine learning techniques. In Section 3, we compare the performance and the computational requirements of the techniques based on experiments on the same dataset. We discuss the results in Section 4, and draw conclusions and indicate directions for future research in Section 5.

## 2. Material and Methods

### 2.1. Data Acquisition

We used the six MTw sensor units that are part of the MTw Software Development Kit manufactured by Xsens Technologies [27]. Each unit comprises three tri-axial devices (accelerometer, gyroscope, and magnetometer/compass) with respective ranges of ±120 m/s^2^, ±1200^°^/s, and ±1.5 Gauss, and an atmospheric pressure meter with 300–1100 hPa operating range, which we did not use. We calibrated the sensors before each volunteer began the experiments and captured and recorded raw motion data with a sampling frequency of 25 Hz. Acceleration, rate of turn, and the strength of the Earth's magnetic field along three perpendicular axes (*x, y, z*) were recorded for each unit. Measurements were transmitted over an RF connection (ZigBee) to Xsens' Awinda Station connected to a remote PC with a USB interface.

### 2.2. Experimental Procedure

**Figure 1.**
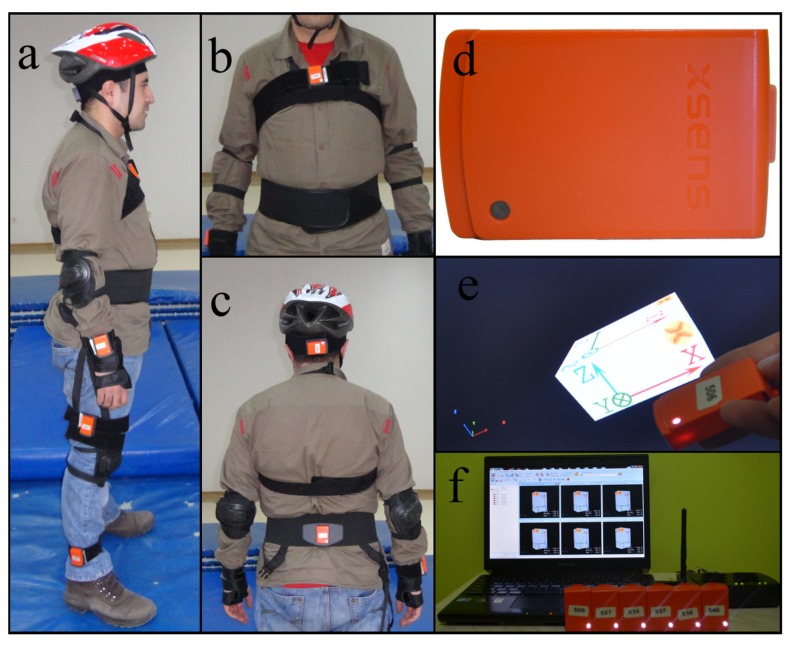
(**a**–**c**) The configuration of the six MTw units on a volunteer's body; (**d**) single MTw unit, encasing three tri-axial devices (accelerometer, gyroscope, and magnetometer) and an atmospheric pressure sensor; (**e**) the three perpendicular axes of a single MTw unit; (**f**) remote computer, Awinda Station and the six MTw units.

We followed the guidelines provided in [23] for designing fall experiments. With Erciyes University Ethics Committee approval, seven male (24 ± 3 years old, 67.5 ± 13.5 kg, 172 ± 12 cm) and seven female (21.5 ± 2.5 years old, 58.5 ± 11.5 kg, 169.5 ± 12.5 cm) healthy volunteers participated in the study with informed written consent. We performed the tests at Erciyes University Clinical Research and Technology Center. We fitted the six wireless sensor units tightly with special straps to the subjects' head, chest, waist, right wrist, right thigh, and right ankle (Figure 1). Unlike cabled systems, wireless data acquisition allows users to perform motions more naturally. Volunteers wore a helmet, wrist guards, knee and elbow pads, and performed the activities on a soft crash mat to prevent injuries, each trial lasting about 15 s on the average.

A set of trials consists of 20 fall actions and 16 ADLs (Table 1) adopted from [23]; the 14 volunteers repeated each set five times. We thus acquired a considerably diverse dataset comprising 1400 falls (20 tasks × 14 volunteers × 5 trials) and 1120 ADLs (16 tasks × 14 volunteers × 5 trials), resulting in 2520 trials. Many of the non-fall actions included in our dataset are high-impact events that may be easily confused with falls. Such a large dataset is useful for testing/validating fall detection and classification algorithms.

### 2.3. Feature Selection and Reduction

Earlier studies on fall detection mostly use simple thresholding of the sensory outputs (e.g., accelerations, rotational rates) because of its simplicity and low processing time. This approach is not sufficiently robust or reliable because there are different fall types and their nature shows variations for each individual. Furthermore, certain ADLs can be easily confused with falls. For improved robustness, we consider additional features of the recorded signals. The total acceleration of the waist accelerometer is given by:

(1) $$A_{T}=\sqrt{A_{x}^{2}+A_{y}^{2}+A_{z}^{2}}$$

where *A_x_, A_y_*, and *A_z_* are the accelerations along the *x, y*, and *z* axes, respectively. We first identify the time index corresponding to the peak *A_T_* value of the waist accelerometer in each record. Then, we take the two-second intervals (25 Hz × 2 s = 50 samples) before and after this point, corresponding to a time window of 101 samples (50 + *A_T_* index + 50) and ignore the rest of the record. Data from the remaining axes of each sensor unit are also reduced in the same way, considering the time index obtained from the waist sensor as reference, resulting in six 101 × 9 arrays of data. Each column of data is represented by an *N* × 1 vector s = [*s*_1_, *s*_2_,…, *s_N_*]*^T^*, where *N* = 101. Extracted features consist of the minimum, maximum, and mean values, as well as variance, skewness, kurtosis, the first 11 values of the autocorrelation sequence, and the first five peaks of the discrete Fourier transform (DFT) of the signal with the corresponding frequencies:

(2) $$\begin{array}{lll} \text{mean}(\text{s}) & =\mu =\frac{1}{N}\overset{N}{\underset{n=1}{\text{∑}}}s_{n}\\ \text{variance}(\text{s}) & =\sigma^{2}=\frac{1}{N}\overset{N}{\underset{n=1}{\text{∑}}}{\left(s_{n}-\mu \right)}^{2}\\ \text{skewness}(\text{s}) & =\frac{1}{N\sigma^{3}}\overset{N}{\underset{n=1}{\text{∑}}}{\left(s_{n}-\mu \right)}^{3}\\ \text{kurtosis}(\text{s}) & =\frac{1}{N\sigma^{4}}\overset{N}{\underset{n=1}{\text{∑}}}{\left(s_{n}-\mu \right)}^{4}\\ \text{autocorrelation}(\text{s}) & =\frac{1}{N-\Delta }\overset{N-\Delta -1}{\underset{n=0}{\text{∑}}}\left(s_{n}-\mu \right)\left(s_{n-\Delta }-\mu \right) & \Delta =0,1,\ldots ,N-1\\ {\text{DFT}}_{q}(\text{s}) & =\overset{N-1}{\underset{n=0}{\text{∑}}}s_{n}e^{-\frac{j^{2}\pi qn}{N}}q=0,1,\ldots ,N-1 \end{array}$$

Here, DFT*_q_*(s) is the *q*th element of the 1-D N-point DFT. We performed feature extraction for the 15,120 records (36 motions × 14 volunteers × 5 trials × 6 sensors). The first five features extracted from each axis of a sensor unit are the minimum, maximum, mean, skewness, and kurtosis values. Because each unit contains nine axes, 45 features were obtained (9 axes × 5 values). Autocorrelation produces 99 features (9 axes × 11 features). DFT produces 5 frequency and 5 amplitude values, resulting in a total of 90 features (9 axes × 10 values). Thus, 234 features are extracted from each sensor unit in total (45 + 99 + 90), resulting in a feature vector of dimension 1404 × 1 (=234 features x 6 sensors) for each trial.

**Figure 2.**
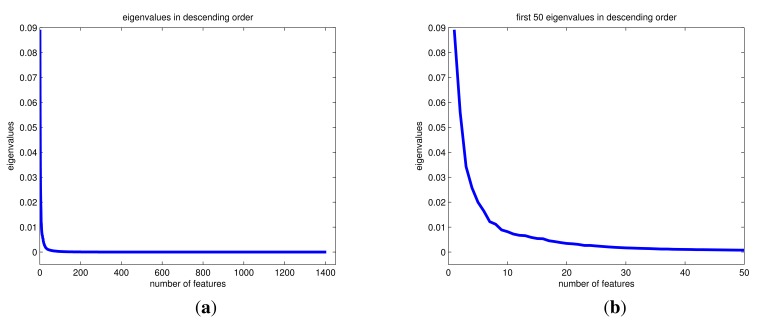
(**a**) All eigenvalues (1404) and (**b**) the first 50 eigenvalues of the covariance matrix sorted in descending order.

Because the initial set of features was quite large (1404) and not all features were equally useful in discriminating between the falls and ADLs, to reduce the computational complexity of training and testing the classifiers, we reduced the number of features from 1404 to *M* = 30 through principal component analysis (PCA) [28] and normalized the resulting features between 0 and 1. PCA is a transformation that finds the optimal linear combinations of the features, in the sense that they represent the data with the highest variance in a feature subspace, without taking the intra-class and inter-class variances into consideration separately. The reduced dimension of the feature vectors is determined by observing the eigenvalues of the covariance matrix of the 1404 × 1 feature vectors, sorted in Figure 2a in descending order. The largest 30 eigenvalues constitute 72.38% of the total variance of the principal components and account for much of the variability of the data. The 30 eigenvectors corresponding to the largest 30 eigenvalues (Figure 2b) are used to form the transformation matrix, resulting in 30 × 1 feature vectors.

### 2.4. Classification Using Machine Learning Techniques

A reliable fall detection system requires well-designed, fast, effective, and robust algorithms to make a binary decision on whether a fall has occurred. Its performance can be measured by the following success criteria:

*Sensitivity* (*Se*) is the capacity of the system to detect falls and corresponds to the ratio of true positives to the total number of falls:
  (3) $$Se=\frac{TP}{TP+FN}\times 100$$
*Specificity* (*Sp*) is the capacity of the system to detect falls only when they occur:
  (4) $$Sp=\frac{TN}{TN+FP}\times 100$$
*Accuracy* (*Acc*) corresponds to the correct differentiation between falls and non-falls:
  (5) $$Acc=\frac{TP+TN}{TP+TN+FP+TN}\times 100$$

Here, TP (a fall occurs; the algorithm detects it), TN (a fall does not occur; the algorithm does not detect a fall), FP (a fall does not occur but the algorithm reports a fall), and FN (a fall occurs but the algorithm misses it) are the numbers of true positives and negatives, and false positives and negatives, respectively. Obviously, there is an inverse relationship between sensitivity and specificity For instance, in an algorithm that employs simple thresholding, as the threshold level is decreased, the rate of FN decreases and the sensitivity of the algorithm increases. On the other hand, FP rate increases and specificity decreases. As the threshold level is increased, the opposite happens: sensitivity decreases and specificity increases. Based on these definitions, FP and FN ratios can be obtained as:

$$\begin{array}{c} \text{FP ratio}=1-Sp\\ \text{FN ratio}=1-Se \end{array}$$

In this study, we consider falls with ADLs because falls typically occur unexpectedly while performing daily activities. An ideal fall detection system should especially be able to distinguish between falls and ADLs that can cause high acceleration of body parts (e.g., jumping, sitting down suddenly). The algorithms must be sufficiently robust, intelligent, and sensitive to minimize FPs and FNs. False alarms (FPs) caused by misclassified ADLs, although a nuisance, can be canceled by the user. However, it is crucial not to misclassify falls as some other activity. FNs, which indicate missed falls, must be avoided by all means, since user manipulation may not be possible if a fall results in physical and/or mental impairment. For example, long periods of inactivity (such as those that may occur after a fall) may be confused with the state of sleeping or resting.

We distinguish falls from ADLs with six machine learning techniques and compare their performances based on their sensitivity, specificity, accuracy, and computational complexity. In training and testing, we randomly split the dataset into *p* = 10 equal partitions and employ *p*-fold cross validation. We use *p* − 1 partitions for training and reserve the remaining partition for testing (validation). When this is repeated for each partition, training and validation partitions cross over in *p* successive rounds and each record in the dataset gets a chance of validation.

#### 2.4.1. The *k*-Nearest Neighbor Classifier (k-NN)

The *k*-NN method classifies a given object based on the closest training object(s) [28]. Class decision is made by majority voting from among a chosen number of nearest neighbors *k*, where *k* > 0. There is no standard value for *k* because the k-NN algorithm is sensitive to the local data structure. Smaller *k* values increase the variance and make the results less stable, whereas larger *k* values increase the bias but reduce the sensitivity. Therefore, the proper choice of *k* depends on the particular dataset. In this work, we determined the value of *k* experimentally as *k* = 7, based on our dataset.

#### 2.4.2. The Least Squares Method (LSM)

In LSM, two average reference vectors are calculated for the two classes that correspond to falls and ADLs [28]. A given test vector x = [*x*_1_,…,*x_m_*]*^T^* is compared with each reference vector **r***_i_* = [*r_i_*_1_, …*, r_i__M_*]*^T^, i* = 1, 2 by calculating the sum of the squared differences between them:

(6) $${ɛ}_{i}^{2}=\overset{M}{\underset{m=1}{\text{∑}}}{\left(x_{m}-r_{im}\right)}^{2}$$

The class decision is made by minimizing ${ɛ}_{i}^{2}$.

#### 2.4.3. Support Vector Machines (SVM)

The initial set of coefficients and kernel models affect the classification outcome of SVMs. The training data (x*_j_*, *l_j_*), *j* = 1,…, *J* is of length *J*, where *x_j_* ∈ ℝ*^N^* and the class labels are *l_j_* ∈ {1, −1} for the two classes (falls and ADLs). We used a radial basis kernel function *K*(x, x_j_) = *e*^−*γ*|x−x*_j_*|^2^^, where *γ* = 0.2, with a library for SVM, called LIBSVM toolbox in the MATLAB environment [29].

#### 2.4.4. Bayesian Decision Making (BDM)

BDM is a robust and widely used approach in statistical pattern classification. We use the normal density discriminant function for the likelihood in BDM, where the parameters are the mean ***µ*** and the covariance matrix **C** of the training vectors for each class. These are calculated based on the training records of the two classes and are constant for each fold. A given test vector x is assigned to the class with the larger likelihood calculated as follows [28]:

(7) $${\text{L}}_{\left(\mathrm{class}\;i\right)}=-\frac{1}{2}\left\{{\left(\text{x}-\mu_{i}\right)}^{T}C_{i}^{-1}\left(\text{x}-\mu_{i}\right)+\log\left[\det\left({\text{C}}_{i}\right)\right]\right\}\;i=1,2$$

#### 2.4.5. Dynamic Time Warping (DTW)

DTW provides a measure of the similarity between two time sequences that may vary in time or speed [30]. The sequences are warped nonlinearly in time to find the least-cost warping path between the test vector and the stored reference vectors. Typically, the Euclidean distance is used as a cost measure between the elements of the test and reference vectors. DTW is employed in applications such as automatic speech recognition to handle different speaking speeds, signature and gait recognition, ECG signal classification, fingerprint verification, word spotting in handwritten historical documents on electronic media and machine-printed documents, and face localization in color images. Here, DTW is used for classifying feature vectors of different activities extracted from the signals of motion sensor units.

#### 2.4.6. Artificial Neural Networks (ANNs)

ANNs are comprised of a set of independent processing units that receive inputs through weighted connections [31]. We implemented a three-layer ANN with 30 neurons each in the input and the hidden layers, and a single neuron at the output layer. In the hidden layer, we use the sigmoid activation function. At the output neuron, we use the purelin linear activation function, which makes the class decision according to the rule:

If OUT≥0.5then ADL,else fall

We created the ANN using the Neural Networks Toolbox in the MATLAB environment and trained it with the Levenberg-Marquardt algorithm.

## 3. Results

**Figure 3.**
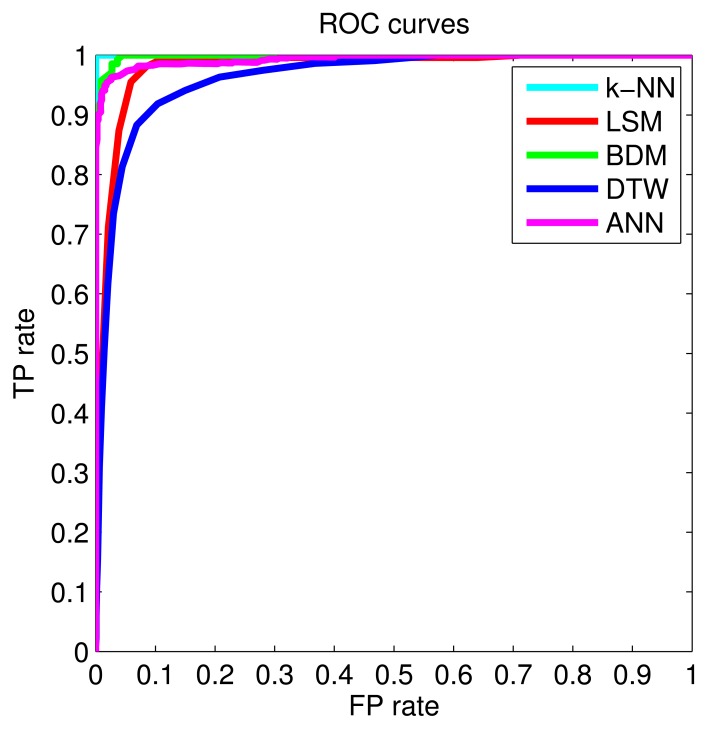
ROC curves for some of the classifiers.

The framework used for the study is subject independent; the classifiers considered here were used to process the complete dataset, instead of designing different classifiers for each subject. We present the performance comparison of the six classifiers in Table 2. The *k*-NN classifier gives the best accuracy (99.91%), followed by LSM, SVM, BDM, DTW, and ANN. The *k*-NN has 100% sensitivity, indicating that falls are not missed with this method; however, two to three ADLs were misclassified over 2520 trials in 10 rounds (Table 3). The average accuracies and standard deviations of the classifiers over 10 rounds are provided in Table 3, where we observe the similarity of the results in each round, indicating their repeatability. Because the *k*-NN classifier and LSM do not miss any falls, we consider them both reliable classifiers. ROC curves for the classifiers are depicted in Figure 3.

We compare the computational requirements of the six machine learning techniques in the last two rows of Table 2 in terms of the training and testing times required for a single fold of the dataset that contains 252 feature vectors. We implemented the algorithms in a MATLAB 7.7.0 environment on a Windows 7 computer with a 2.67 GHz quad core 64-bit Intel Core i5 processor and 4 GB of RAM. In terms of the required training time, the classifiers can be sorted as BDM, LSM, DTW, k-NN, SVM, and ANN in increasing order. In terms of the testing time, the order is ANN, SVM, LSM, BDM, k-NN, and DTW.

## 4. Discussion

The availability of standardized open databases allows researchers to compare their results with those of others. Diversity of the subjects, activity spectrum, and the number of trials are important factors in constructing a database. When a limited number of activities that are easy to discriminate between are performed by a small number of subjects, it may be possible to achieve very high accuracies. However, such performance may not be maintained when the set of activities is broadened or new subjects participate in the tests. Although some studies with very high (∼100%) sensitivity and specificities exist [32,33], the performance of these algorithms degrades when implemented in the real world under realistic conditions and with new users. There are many academic works with promising results but no reliable off-the-shelf product on the market.

**Figure 4.**
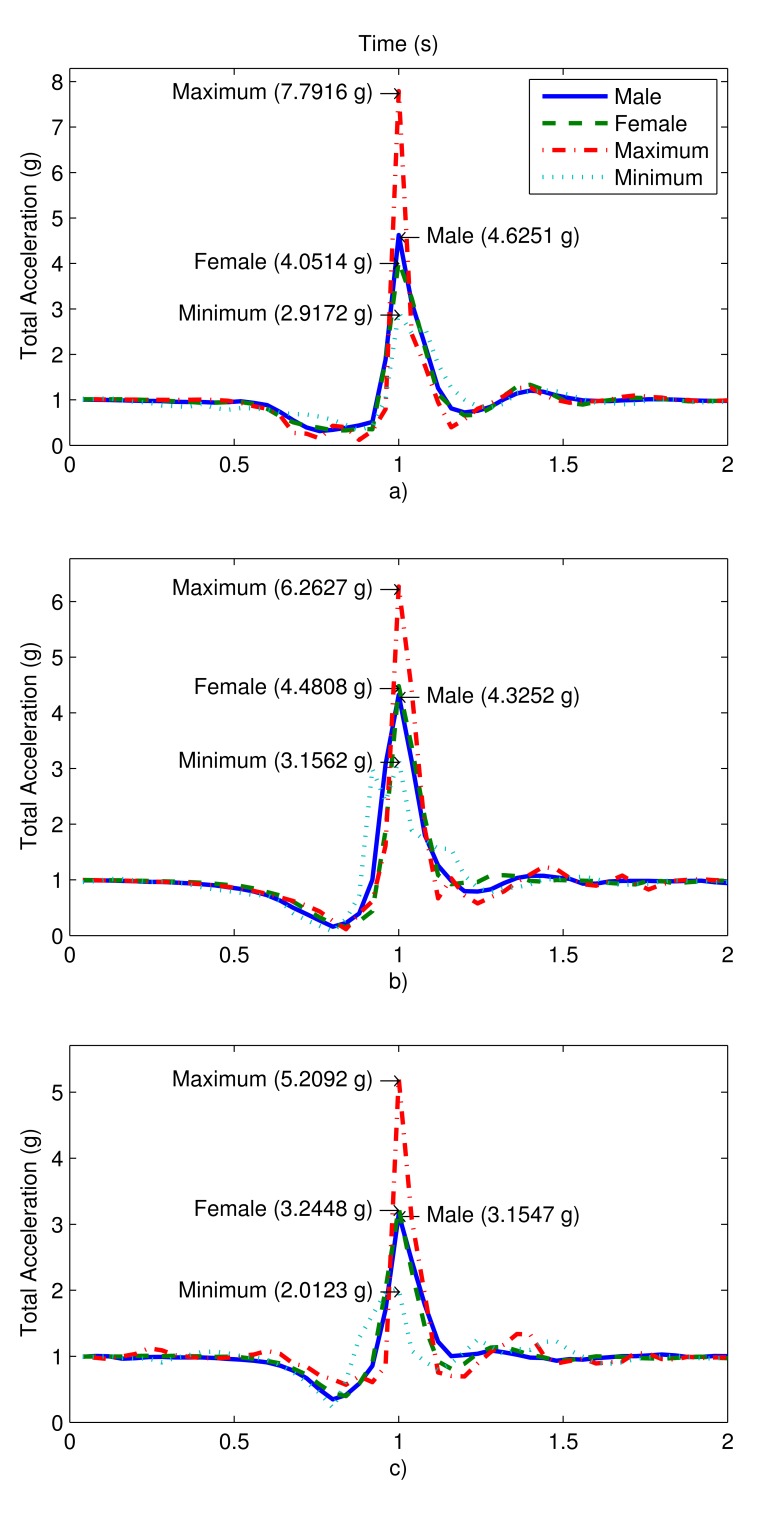
Total acceleration of the waist sensor during the fall actions: (**a**) back sitting; (**b**) back lying; and (**c**) rolling out of bed. The average total acceleration for female/male volunteers and the overall minimum/maximum total acceleration values that occurred during the experiments are shown.

The ADLs that we recorded in this study and included in our dataset are a subset of real-world ADLs, many of which are high-impact events that may be easily confused with falls. Since laboratory-recorded ADLs/falls and those that occur in a natural setting may have some differences, we compared the average and peak acceleration values of the voluntary falls that we recorded, with those in [17], where some involuntary falls by the elderly are recorded. Figure 4 shows sample signals recorded by the waist sensor in our experiments (which is also the location of the sensor in [17]). Back sitting, back lying, and rolling out of bed (Table 1; fall actions 9, 10, and 20, respectively) recordings are illustrated, with average values for female/male volunteers over 35 (= 7 subjects × 5 trials) fall actions each and the minimum/maximum total acceleration. The minimum/maximum values are determined over all records and may belong to a female or a male volunteer. We observe that for a given type of fall, features of the signals recorded from voluntary and involuntary falls are similar in nature. The average duration of the impact from the maximum to the minimum value of total acceleration in both fall types (voluntary and involuntary) is about 0.2 s. Thus, our experimental records are consistent with involuntary falls recorded in an independently conducted study.

Our approach would be applicable to real-world settings where continuous data streams of indeterminate length, containing multiple activities, are recorded. If the data stream contains falls in between a sequence of ADLs, the multiple acceleration peaks can be easily identified. The signal pattern in the time window around each peak can then be processed with machine learning techniques to evaluate if it indeed corresponds to a fall. In real-world testing, we expect our system to give slightly lower accuracies than under laboratory conditions.

The algorithms can be easily embedded into portable devices or accessories carried on the body that can be connected to a telephone network [34]. This feature will allow prompt medical attention, improve the safety, independence, and quality of living of those in fall risk groups, and contribute to the economy by reducing the costs of medical healthcare.

## 5. Conclusions

We employ six classifiers based on machine learning to distinguish between falls and ADLs using previously proposed, standardized experimental procedures. We compare the performance and computational requirements of the machine learning techniques based on the same dataset and achieve accuracies above 95%. The repeatability of the results over the 10 runs indicates the robustness of the classifiers. The *k*-NN and LSM methods do not miss any falls; thus, we consider them reliable classifiers. These classifiers also have acceptable computational requirements for training and testing, making them suitable for real-time applications. The fact that we use standardized experimental procedures to perform a comprehensive set of fall experiments sets an example in the fall detection area. This also makes our approach more applicable to real-world scenarios where data records of indeterminate length, containing multiple activities in sequence, are recorded. We plan to test the system with continuous data streams acquired from falls and ADLs. To enable comparison among the algorithms developed in different studies, we intend to make our dataset publicly available at the University of Irvine Machine Learning Repository [35]. Our daily and sports activities dataset is already available at the same website [36]. In our current work, we are investigating which of the six motion sensor units and which axes of these sensors are most useful in activity and fall detection [37]. Incorporating information from biomedical sensors for vital signs and audio sensors may further improve the robustness of our fall detection system. Our ongoing work considers embedding fall detection algorithms to a mobile device (e.g., a smartphone) to be worn around the waist level.

## Figures and Tables

**Table 1.** Fall and non-fall actions (ADLs) considered in this study.

**Fall Actions**		

**#**	**Label**	**Description**
**1**	front-lying	from vertical falling forward to the floor
**2**	front-protecting-lying	from vertical falling forward to the floor with arm protection
**3**	front-knees	from vertical falling down on the knees
**4**	front-knees-lying	from vertical falling down on the knees and then lying on the floor
**5**	front-right	from vertical falling down on the floor, ending in right lateral position
**6**	front-left	from vertical falling down on the floor, ending in left lateral position
**7**	front-quick-recovery	from vertical falling on the floor and quick recovery
**8**	front-slow-recovery	from vertical falling on the floor and slow recovery
**9**	back-sitting	from vertical falling on the floor, ending sitting
**10**	back-lying	from vertical falling on the floor, ending lying
**11**	back-right	from vertical falling on the floor, ending lying in right lateral position
**12**	back-left	from vertical falling on the floor, ending lying in left lateral position
**13**	right-sideway	from vertical falling on the floor, ending lying
**14**	right-recovery	from vertical falling on the floor with subsequent recovery
**15**	left-sideway	from vertical falling on the floor, ending lying
**16**	left-recovery	from vertical falling on the floor with subsequent recovery
**17**	syncope	from standing falling on the floor following a vertical trajectory
**18**	syncope-wall	from standing falling down slowly slipping on a wall
**19**	podium	from vertical standing on a podium going on the floor
**20**	rolling-out-bed	from lying, rolling out of bed and going on the floor

**Non-Fall Actions (ADLs)**		

**#**	**Label**	**Description**

**21**	lying-bed	from vertical lying on the bed
**22**	rising-bed	from lying to sitting
**23**	sit-bed	from vertical to sitting with a certain acceleration onto a bed (soft surface)
**24**	sit-chair	from vertical to sitting with a certain acceleration onto a chair (hard surface)
**25**	sit-sofa	from vertical to sitting with a certain acceleration onto a sofa (soft surface)
**26**	sit-air	from vertical to sitting in the air exploiting the muscles of legs
**27**	walking-fw	walking forward
**28**	jogging	running
**29**	walking-bw	walking backward
**30**	bending	bending about 90 degrees
**31**	bending-pick-up	bending to pick up an object on the floor
**32**	stumble	stumbling with recovery
**33**	limp	walking with a limp
**34**	squatting-down	squatting, then standing up
**35**	trip-over	bending while walking and then continuing walking
**36**	coughing-sneezing	coughing or sneezing

**Table 2.** Comparison of the results and the computational requirements of the six machine learning techniques in terms of the training and testing times for a single fold (P: positive, N: negative).

		**k-NN**			**LSM**			**SVM**			**BDM**			**DTW**			**ANN**		

		**Confusion Matrices**																	

		**P**		**N**	**P**		**N**	**P**		**N**	**P**		**N**	**P**		**N**	**P**		**N**
**True**	**P**	1400		0	1400		0	1393.9		6.1	1398		2	1381.4		18.6	1364.6		35.4
	**N**	2.3		1117.7	8.7		1111.3	7		1113	16.7		1103.3	35.5		1084.5	73.5		1046.5

***Se*** (%)		100			100			99.56			99.86			98.67			97.47		
***Sp*** (%)		99.79			99.22			99.38			98.51			96.83			93.44		
***Acc*** (%)		99.91			99.65			99.48			99.26			97.85			95.68		

**Computational Time (ms)**																				

**Training**		318.2			2.2			893.7			1.9			2.5			10,089.0		
**Test**		76.6			32.7			16.2			72.6			33,816.6			13.5		

**Table 3.** Classifier results over 10 successive rounds. AVG: average, STD: standard deviation (continued).

**Run**	**1**	**2**	**3**	**4**	**5**	**6**	**7**	**8**	**9**	**10**	**AVG**	**STD**
***Se*** (%)	100	100	100	100	100	100	100	100	100	100	100	0
***Sp*** (%)	99.73	99.82	99.82	99.73	99.73	99.82	99.82	99.82	99.82	99.82	99.79	0.0431
***Acc*** (%)	99.88	99.92	99.92	99.88	99.88	99.92	99.92	99.92	99.92	99.92	99.91	0.0192
**TN**	1117	1118	1118	1117	1117	1118	1118	1118	1118	1118	1117.7	0.4830
**FP**	3	2	2	3	3	2	2	2	2	2	2.3	0.4830
**TP**	1400	1400	1400	1400	1400	1400	1400	1400	1400	1400	1400	0
**FN**	0	0	0	0	0	0	0	0	0	0	0	0

**(a) k-NN**																							

**Run**	**1**	**2**	**3**	**4**	**5**	**6**	**7**	**8**	**9**	**10**	**AVG**	**STD**

***Se*** (%)	100	100	100	100	100	100	100	100	100	100	100	0
***Sp*** (%)	99.29	99.29	99.20	99.20	99.20	99.11	99.11	99.38	99.20	99.29	99.22	0.0847
***Acc*** (%)	99.68	99.68	99.64	99.64	99.64	99.60	99.60	99.72	99.64	99.68	99.65	0.0376
**TN**	1112	1112	1111	1111	1111	1110	1110	1113	1111	1112	1111.3	0.9487
**FP**	8	8	9	9	9	10	10	7	9	8	8.7	0.9487
**TP**	1400	1400	1400	1400	1400	1400	1400	1400	1400	1400	1400	0
**FN**	0	0	0	0	0	0	0	0	0	0	0	0

**(b) LSM**																							

**Run**	**1**	**2**	**3**	**4**	**5**	**6**	**7**	**8**	**9**	**10**	**AVG**	**STD**

***Se*** (%)	99.64	99.50	99.64	99.57	99.50	99.57	99.50	99.50	99.57	99.64	99.56	0.0625
***Sp*** (%)	99.46	99.29	98.93	99.46	99.46	99.55	99.29	99.55	99.29	99.46	99.38	0.1882
***Acc*** (%)	99.56	99.40	99.33	99.52	99.48	99.56	99.40	99.52	99.44	99.56	99.48	0.0825
**TN**	1114	1112	1108	1114	1114	1115	1112	1115	1112	1114	1113	2.1082
**FP**	6	8	12	6	6	5	8	5	8	6	7	2.1082
**TP**	1395	1393	1395	1394	1393	1394	1393	1393	1394	1395	1393.9	0.8756
**FN**	5	7	5	6	7	6	7	7	6	5	6.1	0.8756

**(c) SVM**																							

**Run**	**1**	**2**	**3**	**4**	**5**	**6**	**7**	**8**	**9**	**10**	**AVG**	**STD**

***Se*** (%)	99.86	99.86	99.86	99.86	99.86	99.86	99.86	99.86	99.86	99.86	99.86	0
***Sp*** (%)	98.57	98.57	98.48	99.48	98.39	98.57	98.48	98.57	98.48	98.48	98.51	0.0603
***Acc*** (%)	99.29	99.29	99.25	99.25	99.21	99.29	99.25	99.29	99.25	99.25	99.26	0.0268
**TN**	1104	1104	1103	1103	1102	1104	1103	1104	1103	1103	1103.3	0.6749
**FP**	16	16	17	17	18	16	17	16	17	17	16.7	0.6749
**TP**	1398	1398	1398	1398	1398	1398	1398	1398	1398	1398	1398	0
**FN**	2	2	2	2	2	2	2	2	2	2	2	0

**(d) BDM**																							

**Run**	**1**	**2**	**3**	**4**	**5**	**6**	**7**	**8**	**9**	**10**	**AVG**	**STD**

***Se*** (%)	98.71	98.71	98.79	98.79	98.57	98.64	98.79	98.43	98.50	98.79	98.67	0.1313
***Sp*** (%)	97.79	97.96	97.23	97.14	96.61	97.23	96.96	96.61	96.25	96.52	96.83	0.3321
***Acc*** (%)	97.86	97.94	98.10	98.06	97.70	98.02	97.98	97.62	97.50	97.78	97.85	0.1992
**TN**	1084	1086	1089	1088	1182	1089	1086	1082	1078	1081	1084.5	3.7193
**FP**	36	34	31	32	38	31	34	38	42	39	35.5	3.7193
**TP**	1382	1382	1383	1383	1380	1381	1383	1378	1379	1383	1381.4	1.8379
**FN**	18	18	17	17	20	19	17	22	21	17	18.6	1.8379

**(e) DTW**																							

**Run**	**1**	**2**	**3**	**4**	**5**	**6**	**7**	**8**	**9**	**10**	**AVG**	**STD**

***Se*** (%)	97.64	97.93	96.57	98.00	97.29	97.50	97.86	97.00	97.21	97.71	97.47	0.4545
***Sp*** (%)	93.39	93.21	94.11	93.75	92.86	93.57	93.84	94.38	92.86	92.41	93.44	0.6132
***Acc*** (%)	95.73	95.83	95.48	96.11	95.32	95.75	96.07	95.83	95.28	95.36	95.68	0.3048
**TN**	1046	1044	1054	1050	1040	1048	1051	1057	1040	1035	1046.5	6.8678
**FP**	74	76	66	70	80	72	69	63	80	85	73.5	6.8678
**TP**	1367	1371	1352	1372	1362	1365	1370	1358	1361	1368	1364.6	6.3631
**FN**	33	29	48	28	38	35	30	42	39	32	35.4	6.3631

**(f) ANN**																							
